# A review of therapeutic potentials of milk thistle (*Silybum marianum* L.) and its main constituent, silymarin, on cancer, and their related patents

**DOI:** 10.22038/IJBMS.2022.63200.13961

**Published:** 2022-10

**Authors:** Seyyed Amir Emadi, Mahboobeh Ghasemzadeh Rahbardar, Soghra Mehri, Hossein Hosseinzadeh

**Affiliations:** 1 Pharmaceutical Research Center, Department of Pharmacodynamics and Toxicology, School of Pharmacy, Mashhad University of Medical Sciences, Mashhad, Iran; 2 Pharmaceutical Research Center, Pharmaceutical Technology Institute, Mashhad University of Medical Sciences, Mashhad, Iran

**Keywords:** Antineoplastic agents, Anti-oxidants, Milk thistle, Neoplasms, Pharmaceutical – preparations, Plant extracts, Silybum marianum L. Silymarin

## Abstract

For more than 2000 years, *Silybum marianum* L. (milk thistle) has been used for treating different complications such as jaundice, hepatitis, and cancers. It has also been shown that silymarin, a flavonolignan extract of the plant, demonstrates chemopreventive effects against cancers. This patent review presents and discusses recent patents concerning the anticancer effects of *S. marianum* and silymarin. The data were gathered by searching an extensive literature review conducted in Google Scholar, PubMed, Scopus, Google Patent, Patent Scope, and US Patent. Milk thistle and silymarin have been used in a variety of medical, therapeutic, and pharmaceutical fields, according to a large number of documents and patents. Milk thistle and silymarin have been used as complementary treatments for cancers such as skin, prostate, and colorectal cancers, as well as hepatoprotective agents. Silymarin exerts a chemopreventive effect on reactivating cell death pathways by modulation of the antiapoptotic proteins and synergizing with agonists of death domain receptors. Based on the results of these patents, silymarin could be beneficial to oncology patients, especially for the treatment of the side effects of anticancer chemotherapeutics. Following the human propensity to use phytocompounds rather than medicines based on chemical constituents, special attention must be paid to tie the value of milk thistle and silymarin from basic science to clinical applications.

## Introduction

Cumulative epigenetic and genetic changes characterize cancer started in a normal cell (1). Cancer is a leading cause of death worldwide, accounting for nearly 10 million deaths in 2020, or nearly one in six deaths (2). The drugs used in the control and treatment of cancers have relative effectiveness, and due to some side effects on the patients, the need for herbal medicines with fewer side effects seems necessary (3). Research findings indicate that several dietary agents/medicinal plants can be used to prevent cancer and metastasis along with other traditional chemotherapeutic agents (3, 4). Some natural agents are thought to prevent cancer by inducing apoptosis in cancer cells selectively, suppressing growth factors, suppressing growth factors signaling, suppressing cell proliferation, and cancer-promoting angiogenesis, causing mesenchymal-epithelial transformation and disrupting the tumor microenvironment (5). 

*Silybum marianum *L., known as milk thistle, has been used for more than 2000 years for different diseases and has a long history as a medicinal plant in folk medicine against liver disorders, kidney problems, rheumatism, gastronomic disturbances, cardiac disorders, and gall bladder-related disorders such as jaundice, hepatitis, and cirrhosis (3). The plant is a tall, biennial herb that grows up to 5 to 10 feet. It is also characterized by big prickly leaves, large purple flowering heads, and strong spinescent stems. Milk thistle is named for its milky veins on the leaves (6). The plant is indigenous to South and North America, Australia, Southern Europe, North Africa, and some regions in Asia (7). It is traditionally used in Europe as a vegetable in salads, and the seeds are used as a galactagogue for breastfeeding mothers (8). In the book “Al-Hashaish Dioscorides” (which is one of the sources of traditional medicine), this plant is introduced with the name “Harshfbari” and with the image of milk thistle, and its properties and other names are similar to other sources of traditional Iranian medicine (9). *S. marianum* has protective effects against different biological poisons (such as mycotoxins, snake venoms, and bacterial toxins) and chemical poisons (such as metals, fluoride, pesticides, cardiotoxic, neurotoxic, hepatotoxic, and nephrotoxic agents) (10). Silymarin has been shown to significantly reduce lipid peroxidation and exhibit anti-oxidant, antihypertensive, antidiabetic, and hepatoprotective effects (11, 12). Previous research projects disclosed that *S. marianum* reduces the viability, adhesion, and migration of tumor cells by induction of apoptosis and formation of reactive oxygen species (ROS), reducing glutathione levels, B-cell lymphoma 2 (Bcl-2), survivin, cyclin D1, Notch 1 intracellular domain (NICD), as well as enhancing the amount of Bcl-2-associated X protein (Bax) level (13, 14).

**Figure 1:**
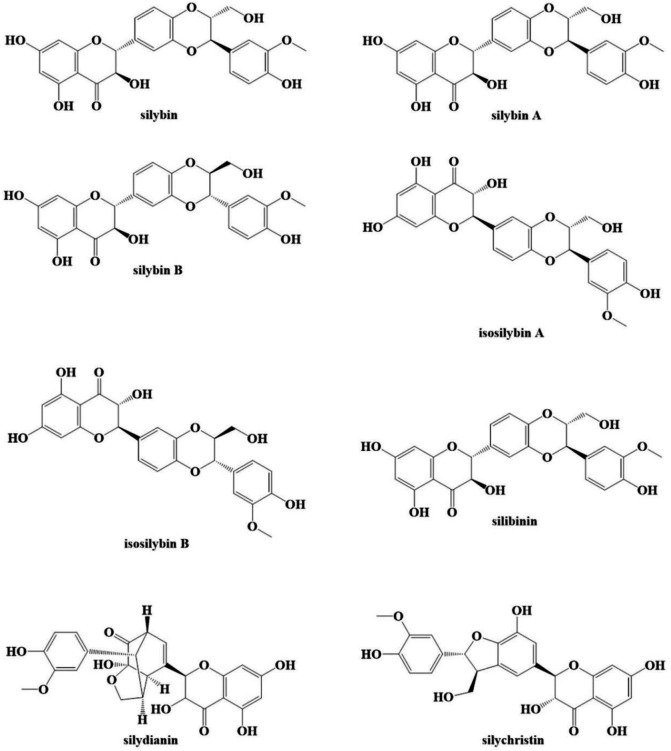
Chemical structures of milk thistle constituents

The main constituent of milk thistle extract is silymarin, which is found in the leaves, seeds, and fruits (15). It contains approximately 70–80% flavonolignans (e.g., silybin, isosilybin, silychristin, isosilychristin, and silydianin), and other flavonoids (e.g., taxifolin, quercetin, and apigenin). The remaining (20–30%) is represented by a relatively undefined polymeric flavonoid fraction. Flavonolignans (except silydianin) exist in silymarin as diastereomeric pairs referred to as A and B in various ratios (16). Silybin is also the main element of silymarin, and it has the highest therapeutic effects compared with other flavonolignans (17) (Figure 1). 

Several studies have indicated that silymarin may suppress the proliferation of different tumor cells, such as prostate (18), breast (19), colon (20), ovary (21), lung (22), and bladder (23). These effects are mediated by cell cycle arrest at the GAP 1/ Synthesis (G1/S)-phase (3), cyclin-dependent kinase inhibitor induction such as p15, p21, and p27 (24), cell-survival kinases, and inflammatory transcription factor inhibition (e.g., nuclear factor-kappa B (NF-κB)) (25, 26), and B-cell lymphoma-extra-large (Bcl-xL)) (25). The suppression of NK-κB-regulated gene products (e.g., cyclooxygenase-2 (COX-2), lipoxygenase (LOX), inducible nitric oxide synthase (iNOS), tumor necrosis factor (TNF), and interleukin-1 (IL-1)) mediates the anti-inflammatory effect of silymarin (18). 

Milk thistle is available as capsules, tablets, tinctures, and intravenous solutions. Its drug interaction is low, and it has no severe effects on cytochromes P-450 (27). Different clinical trials have shown that silymarin is safe for pharmaceutical use and bioavailable (18, 28, 29). Silymarin has demonstrated no significant toxicity in animals (30). Silybin, silydianin, and silychristin have no cytotoxicity or genotoxic effects at 100 μM (31). Silymarin is also safe for humans, hence at therapeutic doses patients demonstrated no negative effects at the high dose of 700 mg, three times a day, for 24 weeks (3). There have been gastrointestinal discomforts such as nausea and diarrhea (32). 

To highlight the importance of the beneficial pharmacological properties of this herb and attract scientists and pharmacists’ attention to formulate more effective medications using medicinal herbs, the preventive and therapeutic potentials of *S. marianum* and its active constituent, silymarin, as well as patents related to their anticancer effects have been summarized in this review.

## Materials and Methods

***Methods***

The present review mainly highlights the published original articles (peer-reviewed and generally unbiased publications) and patents implicating the therapeutic and pharmaceutical properties of milk thistle and silymarin in several cancers from 2010 to 2021. The data (Google Scholar, PubMed, and Scopus) and patents (the US patent, Patentscope, and Google Patent) search have been carried out by searching related keywords including “milk thistle”, “*Silybum marianum”*, “silymarin”, “silibinin”, “silybin”, “silydianin”, “silychristin”, “cancer”, “chemoprevention”, “*in vitro”, “in vivo”*, and “clinical trials”. 

***Effect of milk thistle and silymarin on liver cancer***

Liver cancer is widely found with chronic liver disease and cirrhosis. Hepatocellular carcinoma (HCC), or primary liver cancer, is the fifth most prevalent cancer in men and the seventh most prevalent cancer in women. Besides, it is the third leading cause of death globally due to cancer (33, 34). Despite new achievements in its treatment, liver cancer is still one of the most complicated cancers. In the case of a patient with early HCC, surgery, liver transplantation, and local destructive therapies will suffice. Still, the recurrence of HCC is a severe problem following curative treatment; thus, the incidence rate is above 70% in the fifth year (35). Patients with early and small HCC (<3 cm) who receive surgery cannot have an acceptable 5-year survival rate (47–53%) (35). 

One study evaluated the systemic effect of the silymarin pill (140 mg administered 3 times a day) on hepatotoxicity caused during chemotherapy in 30 breast cancer patients without any metastases. This research reported that one-month treatment with silymarin might significantly reduce the severity of hepatotoxicity in patients who received a doxorubicin/cyclophosphamide-paclitaxel (AC-T) regimen (36).

**Figure 2:**
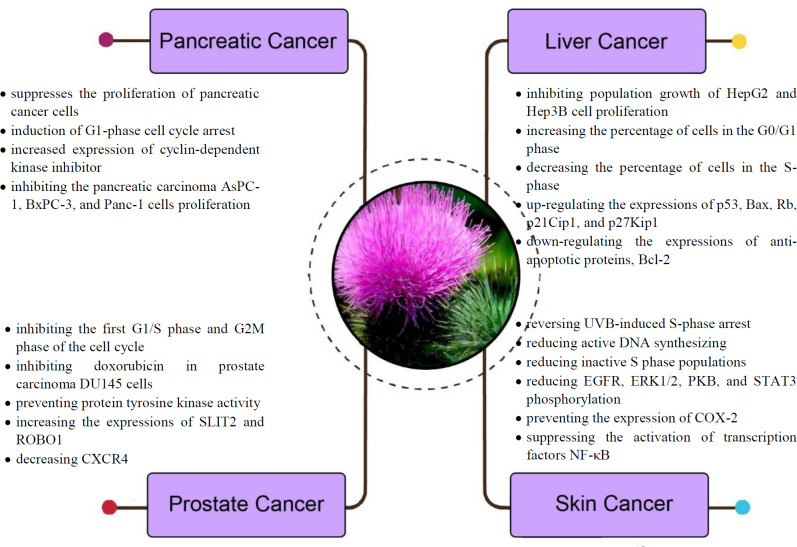
Molecular mechanisms of anti-cancer effects of milk thistle and its constituents

Administration of silymarin at the 1000 ppm dosage level can decrease the total number and multiplicity of macroscopic hepatic nodules in diethylnitrosamine (DENA)-induced hepatocarcinogenesis rats (37). Treatment with silymarin significantly reduced the serum level of alpha-fetoprotein and carcinoembryonic antigen. It also restored the changes in the activities of aspartate transaminase (AST), alanine aminotransferase (ALT), alkaline phosphatase, acid phosphatase, lactate dehydrogenase, γ-glutamyltransferase, and 5′-nucleotidase in the serum and liver of rats with DENA-induced HCC. The histological evaluation of liver samples showed that adduct formation of malondialdehyde–deoxyribonucleic acid (DNA) might be reduced by silymarin administration (38). Severe hyperlipidemia and reduced levels of lipid metabolizing enzymes were seen in rats exposed to DENA. These changes occurred along with up-regulation of hepatic COX-2 expression. Administration of silymarin reduced hyperlipidemia induced by DENA and down-regulated the expression of COX-2 (39) (Figure 2).

***Patents containing milk thistle and silymarin ameliorating liver cancer***

A brief description of patents containing silymarin was summarized in Table 1. A patent formulation (comprising milk thistle, alder tree, tomato, pineapple, broccoli, turnip extracts, etc.) has been shown to improve liver function, decrease alcohol levels in the blood*, *and increase liver function. Milk thistle is used in this formulation because it is a potent anti-oxidant and it can promote protein synthesis, resulting in liver regeneration, preventing the formation of inflammatory prostaglandins and leukotriene, and inhibiting glutathione depletion (40). Another formulation containing milk thistle extract was designed to protect the liver and prevent liver fibrosis. Milk thistle is used to reduce the production of free radicals. It has anti-lipid peroxidation and anti-fibrosis properties (41). Another hepatocyte protective composition containing silymarin and lipoic acid or salt was formulated to be administered as an anti-hepatotoxic drug for hepatocellular protection against hepatitis, cirrhosis, and liver function damage. Silymarin was also used to inhibit hepatocellular toxicity induced by carbon tetrachloride or bromobenzene (42). Moreover, an invention introduced a product comprising a silibinin component for management of viral hepatitis by decreasing the virus load (43). Interventional therapy for liver cancer containing milk thistle was formulated to detoxify and eliminate stagnation, activate blood circulation, remove blood stasis, inhibit the growth of the cancer cells, control disease development, and protect the liver (44). The other invention reveals a liver-protecting compound consisting of milk thistle, *Lathyrus palustris*, Chinese globeflower, licorice roots, and *Melicope patulinervia* with stalks to treat acute and chronic hepatitis, liver fibrosis, and also, to prevent liver cancer (45).

An anti-inflammatory, anti-fibrotic, and anti-oxidant formulation containing silymarin was designed to ameliorate liver fibrosis and possibly reverse established fibrosis in hepatic oxidative stress and cirrhosis. This dietary supplement patent containing milk thistle extract, broccoli extract, and sulforaphane precursor was formulated to enhance liver health, manage nonalcoholic fatty liver disease (NAFLD), and liver cancer, and detoxify the liver by scavenging free radical species, inhibiting the formation of free radicals, preventing membrane lipid peroxidation, enhancing glutathione amount, and chelating iron (46). An invention disclosed an anti-inflammatory and anti-fibrotic anti-oxidant formulation for treating hepatic oxidative stress and cirrhosis. The formulation contains silymarin, *Schisandra chinensis*, *Salvia miltiorrhiza*, oleanolic acid, etc., to slow or probably reverse liver fibrosis by its anti-oxidant property (47). 

***Pharmacological activity and action mechanisms of milk thistle and silymarin on liver cancer***

Silymarin affects liver cancer by several mechanisms. It inhibits the population growth of HepG2, human hepatocellular cancer cells, which results in a rise in the concentration of apoptotic cells. It also may cause a reduction in mitochondrial transmembrane potential due to an increase in cytosolic cytochrome complex (Cyt c) levels. Silymarin does this by up-regulating the expressions of proapoptotic proteins, such as p53, Bax, apoptotic protease-activating factor 1, and caspase-3, and down-regulating the expressions of anti-apoptotic proteins, namely Bcl-2 and survivin, and proliferation-associated proteins, such as proliferating cell nuclear antigen, cyclin D1, c-Myc, and β-catenin (48). Silymarin plays its antiproliferative effect without any changes on nontumor and healthy liver cells. Moreover, silymarin increased the percentage of cells in the gap 0/gap 1 (G0/G1) phase and decreased the percentage of cells in the synthesis (S)-phase, with concomitant up-regulation of retinoblastoma protein (Rb), p53, cyclin-dependent kinase inhibitor 1 (p21^Cip1^), and cyclin-dependent kinase inhibitor 1B (p27^Kip1^) and down-regulation of cyclin D1, cyclin E, cyclin-dependent kinase 4 (CDK4), and phospho-Rb (49). Studies have shown that silibinin inhibited proliferation of Hep3B cells due to simultaneous induction of apoptosis and prevented the accumulation and transcriptional activity of hypoxia-inducible factor 1α exerted strong dephosphorylation of mammalian target of rapamycin (mTOR), and reduced hypoxia-induced vascular endothelial growth factor (VEGF) release (50).

Most of the patents that examined the effect of silymarin on liver diseases were related to reducing the viral load in hepatitis. Fewer patents were for the effect of silymarin on preventing or improving liver damage, and several patents eventually examined the anti-cancer effect of silymarin. Most patents have been limited to oral medications in patients suffering from liver cancer, and silymarin has been used in combination with other therapeutic plants. Silymarin was used for liver disease because of its anti-oxidant effect and anti-fibrosis property. So it can inhibit hepatocellular toxicity and liver regeneration due to promoting protein synthesis. 

One of the strengths of these patents is that, in most of them, silymarin was prescribed to prevent cancer and also to reduce the progression of liver damage to cancer, and the results were satisfactory. But few patents investigate the effect of silymarin on cancer patients. In patents that selected cancer patients as the target population, silymarin inhibited the growth of the cancer cells and controlled disease development.

***Effect of milk thistle and silymarin on pancreatic cancer***

Another fatal cancer is pancreatic cancer, which is mostly associated with a poor prognosis. The disease is one of the most malignant ones, with a challenging onset, late diagnosis, and poor prognosis (51). Inherited genetic change plays a key role in the familial and non-familial occurrences of pancreatic cancer (52). Pancreatic cancers begin from noninvasive masses, usually pancreatic intraepithelial neoplasias. Furthermore, these cancers may develop from intraductal papillary mucinous neoplasms or mucinous cystic neoplasm (53). Only 10 to 15 percent of patients with pancreatic cancer can undergo surgery, and the recurrence rate is also high in these patients after surgical treatment (54). Although new drugs and procedures can help to treat pancreatic cancer patients, the survival rate is still low (55, 56).

Kim *et al*. evaluated the anti-inflammatory effects of silymarin on cerulein-induced acute pancreatitis in mice and reported that silymarin (25 mg/kg, 50 mg/kg, and 100 mg/kg) weakened the severity of acute pancreatitis due to inhibition of p38 mitogen-activated protein kinase (MAPKs) and so it may be a drug for patients with pancreatitis (57). Another investigation demonstrated that silibinin (200 mg/kg daily, administered for 18 days) could induce DNA damage in pancreatic cancer cells and activate caspase 3/7-mediated apoptosis, so it prevented the development of pancreatic cancer cells. Silibinin also reduces cellular myelocytomatosis oncogene (c-MYC) expression, a key regulator of cancer metabolism in pancreatic cancer cells (58). 

***Patents containing milk thistle and silymarin ameliorating pancreatic cancer***

A new mixture containing melatonin, quercetin, zinc, and silymarin was introduced to treat pancreatic cancer. The treatment comprises innovative mixtures of non-chemotherapeutic and nutraceutical medicinal products targeted to prevent metabolic and cancer-supportive signaling pathways and stimulate cancer-suppressive signaling or metabolic pathways (59). Another patent containing silymarin developed a new formulation of a Na^+^/K+-ATPase inhibitor as an oral drug, which was formulated in a pharmaceutically acceptable excipient for pancreatic cancer. Silymarin was used in the mixture as a selective inhibitor of COX-2 to prevent and treat pancreatic cancer (60). A food supplement containing margarine, dairy products, plant juice, fruit juice, pectin, yeast, and milk thistle fruit extract was formulated to strengthen the immune system against cancers, including pancreatic and gallbladder cancer (61).

Another combination of three ingredients containing milk thistle, alpha-lipoic acid, and turmeric was designed to normalize pathological processes associated with diabetes mellitus and liver disorders. Milk thistle (silybin) in this formulation can improve the viability of pancreatic beta-cells through improving oligomerization, inhibiting fibrillation, and reducing beta-cell cytotoxicity of human islet amyloid polypeptide (62).

***Pharmacological activity and action mechanisms of milk thistle and silymarin on pancreatic cancer***

It has been shown that silibinin inhibits the proliferation of pancreatic cancer cells due to the induction of G1 phase cell cycle arrest and increase in the expression of cyclin-dependent kinase inhibitor (p15^INK4B^) (63). Silymarin can also inhibit the production of inflammatory cytokines, such as interleukin-1beta (IL-1β), interferon-gamma (IFNγ), and tumor necrosis factor-alpha (TNF-α), by macrophages and/or T-lymphocytes, which probably initiate the destruction of β-cells in the development of type 1 diabetes (64). Silibinin may cause apoptosis by inhibiting the proliferation of the pancreatic carcinoma AsPC-1, BxPC-3, and Panc-1 cells (65, 66).

As reported, a limited number of patents have examined the effect of silymarin on pancreatic cancer. This may be due to the rarity of pancreatic cancer as well as the late diagnosis of patients with this cancer. These patents show that compounds containing silymarin can prevent and treat pancreatic cancer by stimulating cancer-suppressive signaling or metabolic pathways. Other patents have shown that silymarin is effective in preventing pancreatic cancer by strengthening the immune system against cancer.

***Effect of milk thistle and silymarin on prostate cancer***

The most prevalent non-cutaneous cancer in men is prostate cancer. 1,600,000 new patients are diagnosed every year, and 633,000 patients die annually. Despite the latest developments, cancer is still a major medical problem for men, with overtreatment of inherently benign diseases and inefficient treatment for metastatic prostate cancer (67). 

A study suggested that receiving 570 mg of silymarin daily for six months in patients after radical prostatectomy could decrease low-density lipoprotein (LDL) and total cholesterol (two markers of a serum lipid profile that are associated with prostate cancer progression) (68). In another study, men with benign prostatic hyperplasia received 570 mg of silymarin daily for 6 months and a significant reduction was seen in prostate-specific antigen (PSA) (69). Another study showed that administration of silibinin with ionizing radiation inhibited the proliferation of the endothelial cell as well as reduced the migratory and invasive properties of prostate cancer cells (70). An experimental diet containing 500 ppm silymarin for 40 weeks has shown a significant reduction in the incidence of both prostatic intra-epithelial neoplasm (PIN) and adenocarcinoma. Silymarin suppresses the high proliferative activity of cells started with a carcinogen so that it significantly inhibits proliferating cell nuclear antigen (PCNA) and cyclin D1 labeling indices (71). 

***Patents containing milk thistle and silymarin ameliorating prostate cancer***

A therapeutic composition using silymarin for preventing or treating prostate cancer showed that the formulation inhibited the first gap/synthesis (G1/S) phase and the second gap/mitosis (G2M) phase of the cell cycle. The formulation also prevented protein tyrosine kinase activity (72). Moreover, a patent introduced a method to treat and prevent cell proliferation in prostate cancer and found that five months of combination therapy with silymarin orally at a dosage of 0.1 to 5 g/day led to cancer-free tissue in patients suffering from prostate neoplasia (73). A patent suggested an oral composition with milk thistle extract or milk thistle powder administered to the subject for the treatment of a disease or condition associated with the prostate of a subject (74). A phyto-nutraceutical composition showed that silibinin inhibited prostate tumor development without any significant toxicity, and also prevented the secretion of proangiogenic factors from tumor cells. So it prevents apoptosis of endothelial cells related to disruption of capillary tube formation (75). In another patent, *S. marianum* has been used as an estrogen receptor β-agonist and an inhibitor of PSA for treating prostate cancer (76).

***Pharmacological activity and action mechanisms of milk thistle and silymarin on prostate cancer***

Silibinin strongly increased the growth-inhibitory action of doxorubicin in prostate cancer DU145 cells, which was associated with G2M arrest in cell cycle progression (77). Slit Guidance Ligand 2 (SLIT2)/Roundabout Guidance Receptor 1 (ROBO1) signaling is a very crucial pathway causally implicated in prostate cancer. Silymarin inhibits the expression of C-X-C chemokine receptor type 4 (CXCR4) (a chemokine receptor implicated in cancer progression) and so it increases the expression of SLIT2 and ROBO1 (78). Administration of silymarin causes a decrease in tyrosine phosphorylation of an immediate downstream target of erbB1 with a decrease in its binding to erbB1 (79). Silibinin also has an inhibitory effect on the viability, motility, and adhesion of metastatic PC-3 cells. Thus, silymarin may prevent the metastasis process in prostate cancer (80). 

Although the effects of silymarin on prostate cancer have been studied in a few studies, these few patents have yielded promising results. In two patents, as mentioned, oral silymarin induced cancer-free tissue in patients suffering from prostate neoplasia. In another patent, it inhibited the growth of prostate cancer cells by affecting the secretion of proangiogenic factors. Based on these results, prostate cancer is one of the cancers that should be further studied for the anti-cancer effects of silymarin.

***Effect of milk thistle and silymarin on skin cancer***

Uncontrolled exposure to solar ultraviolet (UV) radiation is the main cause of skin cancer, particularly in the UVC (200–290 nm) and UVB (290–320 nm) ranges. UVB is highly absorbable by cellular DNA in the skin and leads to diverse DNA damage. The most critical DNA lesions caused by photocarcinogenesis are cyclobutane pyrimidine dimers (81). Moreover, oxidative stress (including the generation of free radicals and ROS, and depletion of anti-oxidant machinery) has a role in removing moieties and is a key effect of exposure of the skin to UVB. Such oxidative reactions can lead to DNA damage and many biochemical and molecular events that lead to tumor genesis (39). 

One study evaluated the effect of silibinin on photodamage caused by UVB and showed that silibinin could prevent apoptosis and accelerate the repair of cyclobutane pyrimidine dimers (CPD) induced by UVB. Silibinin changed the arrest of the S phase induced by UVB and decreased active DNA synthesis and inactivated S phase populations (82). 

Silymarin has a significant effect on the treatment and management of the side effects of patients suffering from anticancer radiotherapy and chemotherapy. Evidence from one clinical study showed that it has mainly beneficial effects on hepatotoxicity and radiotherapy-induced skin and mucosa damage at dosages of 160–600 mg daily (83). Another study showed that silymarin could prevent radiotherapy-induced mucositis (84). Karbasforooshan *et al*. have shown that using silymarin 1% gel for 5 weeks delayed the occurrence of radiodermatitis and decreased its severity (85). 

In the Becker-Schiebe study, a silymarin-based cream was evaluated to prevent the occurrence of skin lesions after radiotherapy in cancer patients. After five weeks of radiotherapy, grade 2 toxicity was reported in 9.8% of patients who used silymarin-based cream, in comparison to 52% in the control group. At the end of radiotherapy, grade 3 toxicity occurred in only 2% of the cases, compared with 28% of controls (86). 

***Patents containing milk thistle and silymarin ameliorating skin cancer***

A patent introduced a composition for topical use on mammalian skin to examine the reduction in oxidative stress. The composition increased the cutaneous intrinsic defense mechanism, which improved the general health status and appearance of the skin. The topical use of this preparation also stimulated a DNA repair response, which helped to maintain homeostasis between cellular apoptosis and hyperproliferation. Topical administration also improved skin appearance by improving the thickness of skin and relief of the epidermal rate ridges and/or increasing the density of the collagen network in the dermis (87). A new patent was formulated in South Korea for therapeutic purposes containing silymarin (5.0 wt% of silymarin) as one of the active ingredients to prevent skin damage by inflammation because of 2, 3, 7, 8-tetrachlorodibenzo-p-dioxin, which is an air pollutant. The formulation prevented the expression of cytochrome P450 Family 1 Subfamily A Member 1 (CYP1A1) and COX-2, which are the genes targeted by an aryl hydrocarbon receptor that is activated by representative dioxin, 2, 3, 7, 8-tetrachlorodibenzo-p-dioxin (88). In addition, a pharmaceutical formulation containing *S. marianum *extract at a therapeutic level between 100 mg and 3000 mg reduced inflammation and oxidative stress (89). A mixture containing silymarin was described in another patent, and the results have shown the composition has anti-oxidant activity. The researcher claimed that dermatological agents of this composition improved dermatological conditions due to aging or extrinsic factors such as radiation, sunline, air pollution, wind, dampness, cold, heat, chemical smoking, and smoke (90).

***Pharmacological activities and action mechanisms of milk thistle and silymarin on skin cancer***

Silibinin inhibits the activation of the epidermal growth factor receptor, the downstream adapter protein Shc, and the inhibition of extracellular signal-regulated kinase-1 and -2 (ERK1/2) activation (91). Silibinin also prevents mitogenic signaling by reducing Epidermal Growth Factor Receptor (EGFR), ERK1/2, protein kinase B (PKB), and signal transducer and activator of transcription 3 (STAT3) phosphorylation and suppressed the activation of transcription factors NF-κB and activator protein 1 (AP-1) (92). Silymarin prevents the expression of CYP1A1 and COX-2, which are the genes targeted by an aryl hydrocarbon receptor (86).

Patents concerning the anti-cancer effects of silymarin on skin cancer are more diverse than those concerning other cancers. The patents examined both UV-induced skin cancer and radiotherapy-induced skin cancer (prescribed for the treatment and control of other cancers), but all of these studies aim to prevent skin cancer following sun exposure and radiotherapy, cigarettes, chemical burners, and pollution. In the only study that examined the direct effect of silymarin on skin cancer cells, silymarin significantly inhibited cell growth of basal cell carcinoma. In contrast to other studies in which silymarin was used orally, in a skin cancer patent report, most drugs were in the form of an ointment containing *S. marianum* extract. In the field of skin, silymarin has been studied extensively for cosmetic and rejuvenating purposes, and few cancer-related studies have been performed. 

***Effect of milk thistle and silymarin on fatigue related to cancer***

The fatigue caused by cancer is nothing like the normal fatigue caused by physical activity or stress, which is solvable by resting. Cancer-related fatigue is described as a subjective feeling of tiredness, weakness, or lack of energy that influences daily activities and quality of life, and its prevalence ranges from 25% to 99% depending on the patient, type of therapy, and method of assessment (93, 94). Inflammation may play a role in the etiology of cancer-related fatigue, and peripheral inflammatory cytokines can signal the central nervous system to generate symptoms of fatigue and other behavioral changes via alterations in neural processes (94). A patent preparation in Russia introduced a composition of extracts of common ginseng, pharmaceutical ginger, and milk thistle for fatigue alleviation in cancer patients. The composition was used on male patients with primary tumors of the prostate, pancreas, lung, and colon who experienced fatigue for at least one month with an average life expectancy of eight months minimum. The results have shown that this composition notably improved vitality, peripheral pain, mood, and appetite. These results can be due to a faster release of the stomach contents, less nausea-like feelings, or higher liver function (95).

Bax: Bcl-2-associated X protein; Bcl-2: B-cell lymphoma 2; COX-2: cyclooxygenase-2; EGFR: Epidermal Growth Factor Receptor; ERK1/2: extracellular signal-regulated kinase-1 and -2; G0/G1: gap1/gap2; G1/S: gap1/synthesis; G2M: gap2/mitosis; NF-κB: nuclear factor-kappa B; PKB: protein kinase B; Rb: retinoblastoma; S-phase: synthesis-phase; STAT3: signal transducer and activator of transcription 3; UVB: ultraviolet B

**Table 1:** Brief description of patents disclosing compositions containing silymarin

Patent number	Title	Composition	Development status (description)	Reference
CN1853508A	Composition for the improvement of liver function, reduction of serum ethanol level, and antioxidant activity enhancement.	A composition comprising *Lactobacillus brevis* *HY7401*, *lactobacillus fermenti CS332*, *lactobacillus acidophilus* *CSG*, *bifidobacterium longum* *HY8001*, alder tree extract, Prunella vulgaris extract, milk thistle extract, give birth to beans-rice bran extractive from fermentative, radish Juice (turnip extract), tomato extract, cauliflower extract (broccoli extract), pineapple extract	Improves liver function, reduces oxidation resistance in BAC	(40)
CN108419977A	A kind of *Silybum marianum* Gaertn seed oil solid beverage for relieving alcoholism and protecting liver and preparation method thereof	Milk thistle, *dendrobium candidum*, *hoveniae semoveniae* semen, Radix Ophiopogonis, green tea, Chinese holly Qi, dried longan pulp, Fructus Mume, brown sugar or Aspartame, shell Glycan	Protecting the liver according to specific implementation mode six, takes volunteer's completely not drunk dizziness on the second day, headache afterward, have palpitation, weakness of limbs, irritating symptom, and sober up effect is best	(41)
KR100693613B1	Composition for protecting hepatocyte containing silymarin and lipoic acid or its salts	Lipoic acid or its salt is mixed with silymarin.	Hepatocellular protective effect by silymarin administration on cytotoxicity induced by H2O2	(42)
JP5349486B2	Silibinin component for the treatment of hepatitis	Silibinin (3,5,7-trihydroxy-2- (3- (3-hydroxy-4-methoxy-phenyl) -2- (hydroxymethyl) -2,3-dihydrobenzo [b] - Dioxin-6-yl)	Treatment of hepatitis C in a patient who does not respond to ribavirin/interferon therapy	(43)
CN106377627A	Medicine for assisting interventional therapy of liver cancer.	Milk thistle, fourstamen stephania root, date leaves, *Mussaenda pubescens*, pine needle extract, flos mume, *herba hyperici japonici, *Chinese walnut tree bark, olive leaf extract	Detoxify and eliminate stagnation, activate blood circulation, remove blood stasis, inhibit the growth of the cancer cells, control disease development, and protect the liver	(44)
CN110025686A	A kind of Baoganleifufang and preparation method thereof	of milk thistle, *Lathyrus palustris*, Chinese globeflower, Licorice roots, and *Melicope patulinervia*	Treat acute and chronic hepatitis and liver fibrosis, and also, prevent liver cancer	(45)
US20100086627A1	Methods and formulations for treating chronic liver disease	*Salvia miltiorrhiza, Schisandra chinensis*, oleanolic acid or a pharmaceutically acceptable salt thereof, *Ganoderma lucidum*, silymarin, α-lipoic acid, N-acetyl cysteine, *Picrorhiza kurroa*,	Enhance liver health, manage NAFLD, and liver cancer, and detoxify the liver by scavenging free radical species, inhibiting the formation of free radicals, preventing membrane lipid peroxidation, enhancing glutathione amount, and chelation of iron	(46)
JP2005520787A	Method for increasing glutathione present in cells.	Silymarin, *Schisandra chinensis*, *Salvia miltiorrhiza*, oleanolic acid	Slow or reverse liver fibrosis by its antioxidant property	(47)
WO2012122295A2	Treatment for pancreatic adenocarcinoma and other cancers of epithelial origin.	Melatonin, quercetin, zinc, and silymarin	Prevent metabolic and cancer-supportive signaling pathways and stimulate cancer-suppressive signaling or metabolic pathways	(59)
US20070105790A1	Pancreatic cancer treatment using Na+/K+ ATPase inhibitors.	A composition comprising silibinin	Conjoint therapy with a Na+/K+-ATPase inhibitor for treating a patient having pancreatic cancer	(60)
US6605296B1	Natural substances-based agent.	Margarine, dairy products, plant juice, fruit juice, pectin, yeast, and milk thistle	Strengthen the immune system against cancers, including pancreatic and gallbladder cancer	(61)
CA2983760A1	Three-component herbal formulation for the management of pre-diabetic and diabetic states, and liver diseases.	Milk thistle, alpha-lipoic acid, and turmeric	Improve the viability of pancreatic beta-cells through improving oligomerization, inhibiting fibrillation, and reducing beta-cell cytotoxicity of human islet amyloid polypeptide	(62)
EP1448232B1	Anti-proliferative composition.	Silymarin, lycopene, and genistein	Inhibited the G1/S phase and G2M phase of the cell cycle and prevented protein tyrosine kinase activity	(72)
US20130045179A1	Combination therapy and methods for treatment and prevention of hyperproliferative diseases.	Silymarin is administered orally at a dosage of 0.1 to 5 g/day, preferably 0.4 to 2 g/day	Administration to a patient with cancer of a combination of effective amounts of agents capable of eradicating the neoplastic cells	(73)
TW201402129A	Compositions comprising sulforaphane or a sulforaphane precursor and a milk thistle extract or powder.	Broccoli extract and milk thistle extract	Treatment disease or condition associated with the prostate	(74)
US20080260771A1	Prostate disorder(s) phyto-nutraceutical synergistic composition.	*Panax, Rhapontium, Ganoderma, Grifola, Vitex agnus castus, Arctostaphylos uva ursi, Cucurbita, Pygeum, Selenium, Serenoa, Silybum, Urtica*, Vitamin E, and Zinc	Inhibits the secretion of proangiogenic factors from tumor cells	(75)
EP1904018	Combination preparation, particularly for treating prostate cancer.	*Cimicifuga racemosa, Silybum marianum, Belamcanda chinensis*	Inhibiting PSA	(76)
US20160166626A1	Topical compositions and methods for reducing oxidative stress.	*Camellia oleifera*, *Camellia sinensis*, green tea and white tea; *Wasabia japonica,* *Bacopa monnieri*, *Silybum marianum *	Increased the cutaneous intrinsic defense mechanism	(87)
KR101930311B1	Cosmetic composition containing silymarin for protection of skin damaged by air pollutants.	Silymarin, beeswax, polysorbate, sorbitan sesquioleate, squalane, sorbitan stearate, glyceryl stearate	Preventing skin damage caused by inflammation caused by 2,3,7,8-tetrachlorodibenzo-p-dioxin	(88)
US9265808B2	Compositions for alleviating inflammation and oxidative stress in a mammal.	*Bacopa monnieri, Silybum marianum,* *Withania somnifera, Camellia sinensis, Curcuma longa,* *Centella asiatica, Ginko biloba,* *Aloe vera,* and N-acetyl cysteine.	Reduced the inflammation and oxidative stress	(89)
US20020127256A1	Compositions and methods for treating dermatological disorders	Vitamin A, *Ginko biloba,* silymarin, quercetin compound, vitamin C	Treating a dermatological condition in a patient having skin comprising	(90)

## Conclusion

The protective and healing effects of *S. marianum *and its active constituent, silymarin, besides patents related to their anticancer properties, have been summarized in this review to highlight the significance of the advantageous pharmacological potentials of this herbal medicine and to draw scientists and pharmacists’ attention to formulate more efficient medications with medicinal herbs. Silymarin has a pleiotropic effect on cancer cells with several targets. As stated in this study, several patents have been published regarding the effect of silymarin on liver, pancreas, prostate, and skin cancers, and it has been shown that silymarin has anti-cancer effects. The anti-oxidant and anti-inflammatory effects of silymarin as well as its ability to regulate different proteins and genes causes silymarin to have its anti-chemopreventive action. Therefore, it can play a role in the main stages of carcinogenesis, i.e., the onset, promotion, and progression of the tumor. Silymarin controls these stages by balancing the enzymes of phase I and II metabolism, by progression in the cell cycle, and by inducing apoptosis. Silymarin also reduces the toxic effects on vital organs or healthy cells. It is worth noting that milk thistle and its main constituents in these formulations are likely to have synergistic effects with chemicals included in herb combination formulations, thus the medicinal benefit is not solely due to these compounds. We believe that silymarin, as described in this review, may play a role in adjuvant cancer therapy, and it can be suggested that this natural product can be considered a promising factor in the combined treatment of cancer. However, the number of patent reports of silymarin prescription directly to cancer patients is small, and further investigations are required. Furthermore, future review articles might cover the limitations of the current review and could analyze patents from various companies/assignees as well as from other databases such as the European Patent Office, Trademark Office, Espacenet, Patent Lens, Prior IP, and the World Intellectual Property Organization’s Patent Scope, etc. 

## Authors’ Contributions

SAE Participated in data acquisition, drafting the article, and interpretation; MGR Participated in data acquisition and revision; SM Supervised and revised it critically for important intellectual content; HH Provided conception and design and contributed to administrative, technical, and material support.

## Conflicts of Interest

The authors declare that there are no conflicts of interest.
